# Supplementary material for: HIV–HPV Co-Infection and Identification of Novel High-Risk HPV Among Women at Two Hospital Centers in Cotonou, Republic of Benin
Source: Viruses. 2025 May 16;17(5):714. doi: 10.3390/v17050714 (PMC12115566; doi:10.3390/v17050714)
Supplement: Supplementary file 1 [file viruses-17-00714-s001.zip › viruses-3591910-supplementary.pdf]

Table S1: Primer Sequences and PCR Conditions for House-Keeping Gene (β-globin) Amplification

a. **Primer Sequences**

**Primer ID**	**Primer Sequence**	**Expected band Size**
**PCO3**	5'-CTTCTGACACAACTGTGTTCACTAGC-3'	110 bp
**PCO4**	5'-TCACCACAACTTCATCCACGTTCACC-3'	

Source: Venceslau *et al.,* 2014; Kiani *et al*., 2015

b. **PCR Cycling Conditions**

**Steps**	**Temperature and Duration**	**Number of cycle**
**Initial Denaturation**	95ºC for 5 mins	**1**
**Denaturation**	95ºC for 1 min	
**Annealing**	55ºC for 1 min	**40**
**Extension**	72ºC for 1 min	
**Final extension**	72ºC for 10 mins	**1**

TableS2: Primer sequences and PCR conditions for HPV DNA amplification

a. **Primer sequences for HPV DNA amplification**

Primers	Primer Sequence	Expected band Size (L1 gene)
MY 09	5'-CGTCCMARRGGAWACTGATC-3'	
MY 11	5'-GCMCAGGGWCATAAYAATGG-3'	450bp
PGMY 09 F	5'-CGTCCCAAAGGAAACTGATC-3'	
PGMY 11A	5'-GCACAGGGACATAACAATGG-3'	450bp
GP5+	5'-TTTGTTACTGTGGTAGATACTAC-3'	150bp
GP6+	5'-GAAAAATAAACTGTAAATCATATTC-3'	

b. PCR conditions for first round

Step	Temperature and Duration	Number of cycle
	95ºC for 5 mins	
**Initial Denaturation**	95ºC for 1 min	**1**
**Denaturation**		
**Annealing**		**40**
**Extension**	55ºC for 1 min	
**Final extension**		**1**
	72ºC for 1 min	
	72ºC for 5 mins	

c. PCR conditions for second round

Step	Temperature and Duration	Number of cycle
	95ºC for 5 mins	
**Initial Denaturation**	95ºC for 1 min	**1**
**Denaturation**		
**Annealing**		**40**
**Extension**	42ºC for 1 min	
**Final extension**		**1**
	72ºC for 1 min	
	72ºC for 5 mins	

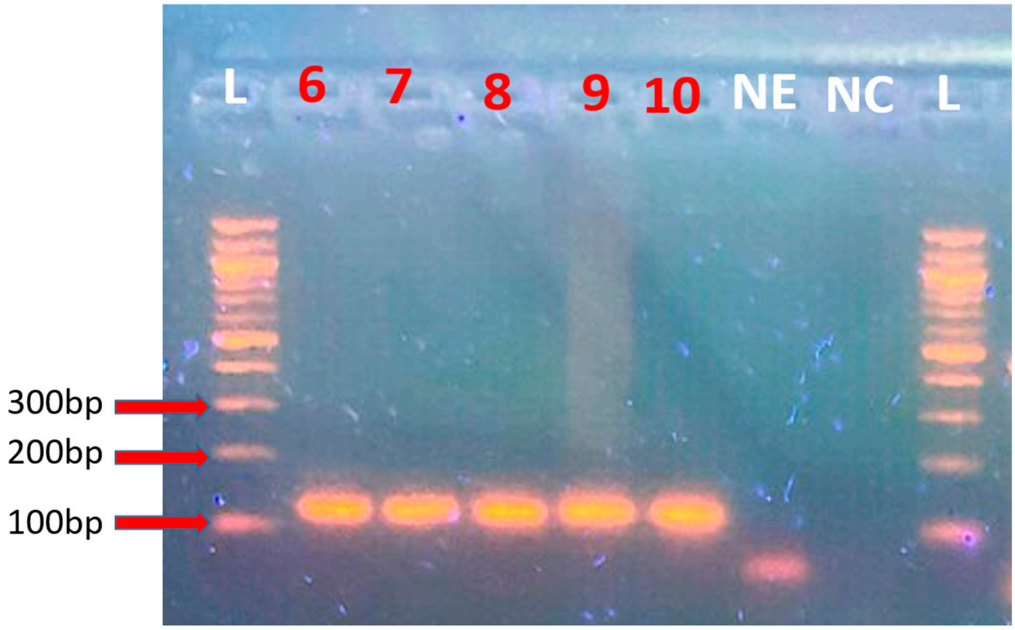

Figure S1: Agarose Gel Representation of β-globin gene (110 bp) PCR

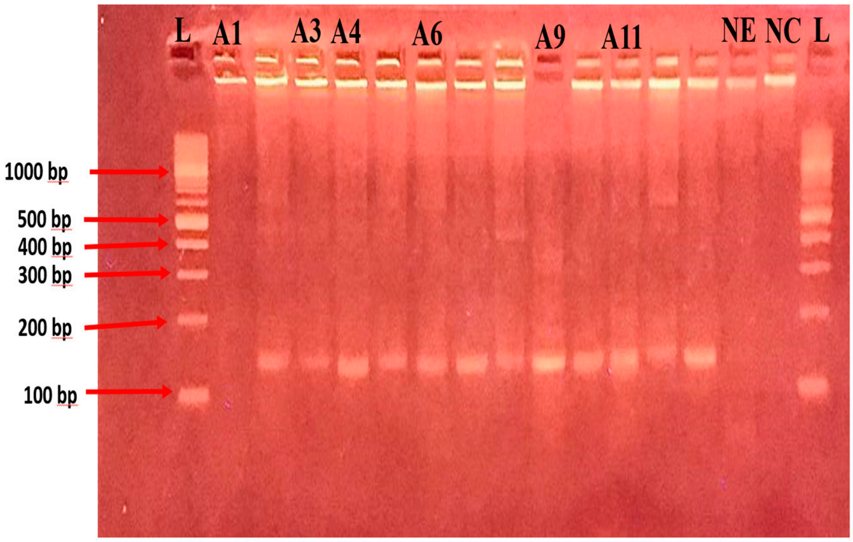

Figure S2: Agarose Gel Representation of the 2nd round PCR (150 bp)

Legend (Figures S1 and S2):

NE: Negative Extract

NC: Negative Control

L: Ladder

a. HPV 45

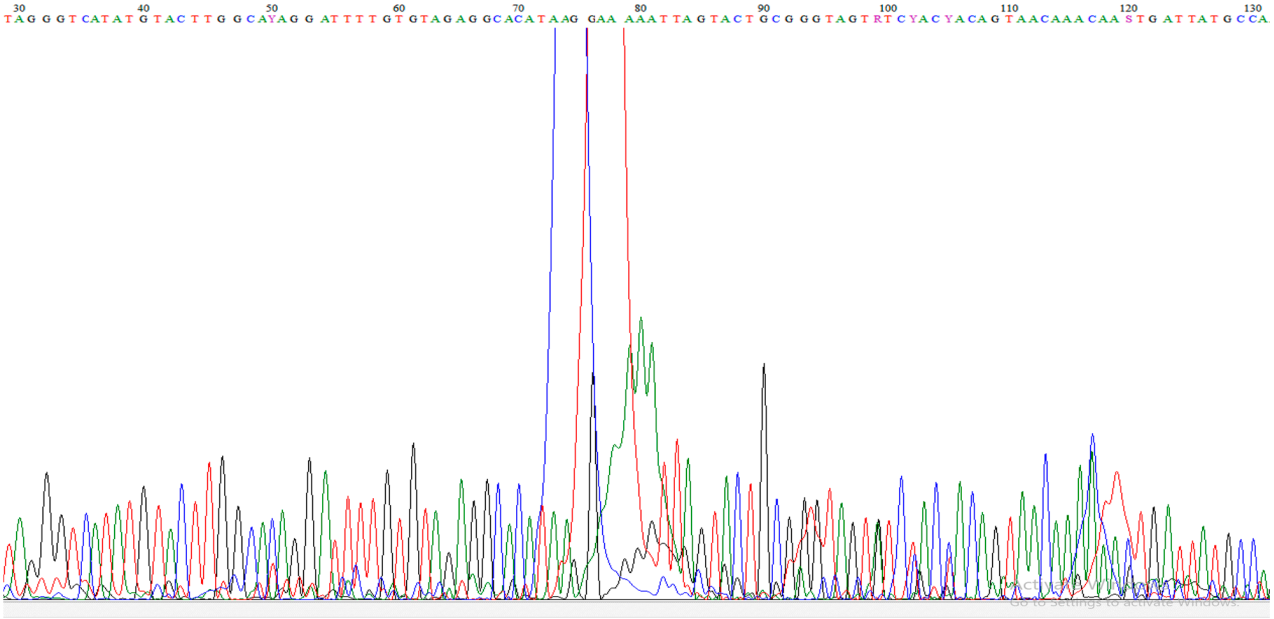

b.HPV 54

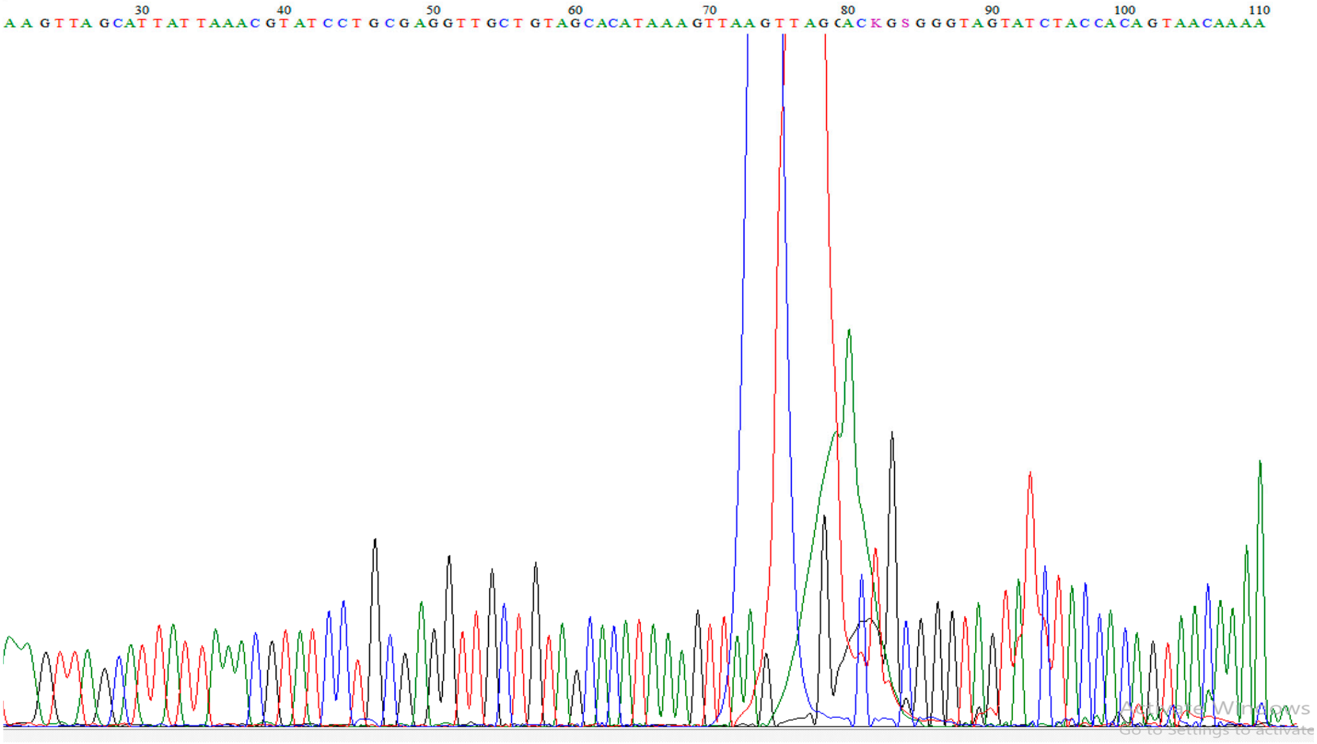

Figure S3: Chromatogram Sequences of HPV 45 and 54 Generated by Sanger Sequencing

a. HPV67

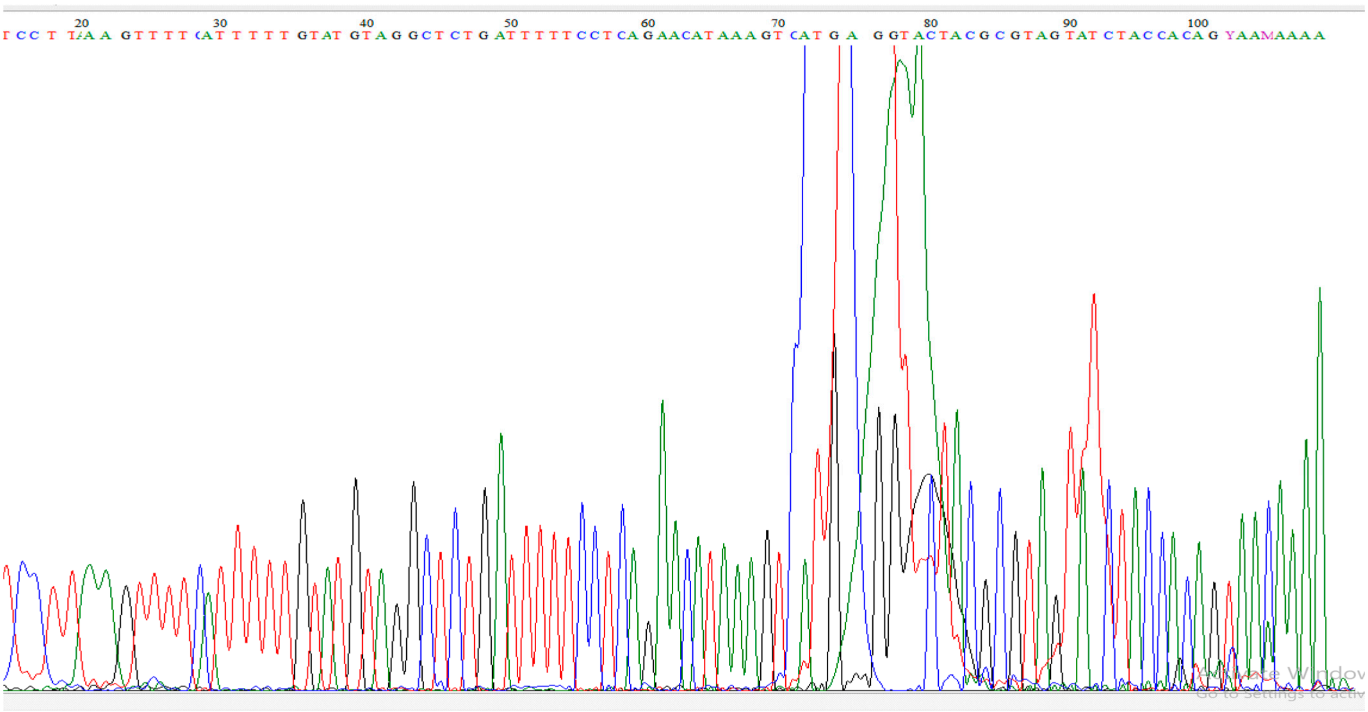

b.HPV 70

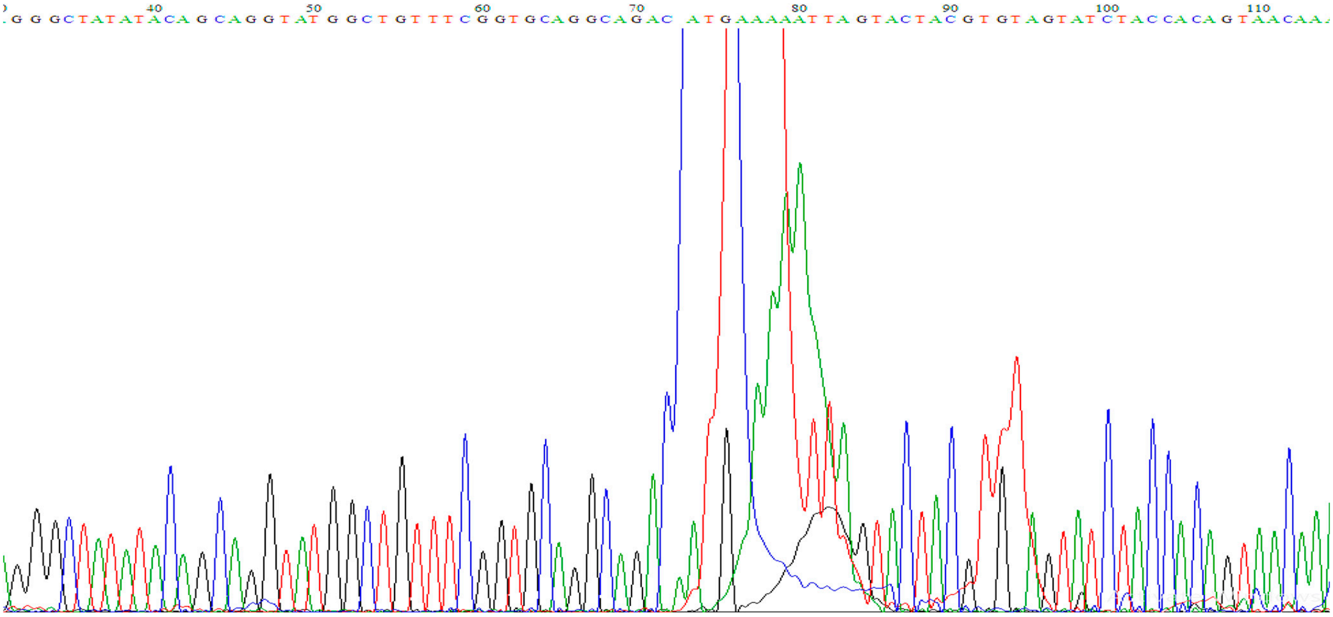

Figure S4: Chromatogram Sequences of HPV 67 and 70 Generated by Sanger Sequencing

Legend (Figures S3 and S4):

Figure S3a: Chromatogram Sequences of HPV 45

Figure S3b: Chromatogram Sequences of HPV 54

Figure S4a: Chromatogram Sequences of HPV 67

Figure S4b: Chromatogram Sequences of HPV 70

These chromatograms show the nucleotide sequence obtained from HPV Sanger sequencing. Each peak corresponds to a DNA base: adenine (green), thymine (red), cytosine (blue), and guanine (black).

Well-defined peaks represent high-confidence base calls, while overlapping peaks may indicate sequencing ambiguities or mixed templates

Table S3: Variable sites in HPV 70 aligned sequences

Position / Sample ID	10	11	43	82	84
Reference	A	A	G/C/S	C	T
GCNM 035	-	T	C	M	G

G/C/S: Variation between reference genomes with G more predominant than C and S

S: Ambiguous nucleotide

-: deletion

Table s4: Variable sites in HPV 70 aligned sequences

Position / Sample ID	8	16	18	19	39	81	83	88
Reference	C	A	A	T	G/A	T	C	C
GCP 083	C	C	-	T	A	A	C	S
GCNM 049	C	T	-	A	A	T	M	S
GCP 051	-	T	A	T	A	A	M	S
GCP 058	C	T	A	T	A	A	C	C

G/A: Variation within reference genomes with G more predominant than A

-: Deletion

Table S5: Variable sites in HPV 82 aligned sequences

Position / Sample ID	69	78	84	91	99	105	108	110	111	117	132	135	138	143	144	146	147
Reference	T	A/G	T/C	A/G	T	A/G	C/T	T	T	C/T	A	A/G	A/G	T	A/T	T	T

GCP 095	A	G	C	A	W	G	-	A	A	C	T	A	G	Y	T	Y	W

Position / Sample ID	7	10	11	12	15	18	21	27	29	36	41	42	43	44	46	59	65
References	A/G	A	C	T	T	T	C	T	G/A	T/C	T	A	A	C	A	T/C	A
GCP 095	G	C	T	G	-	A	T	C	G	T	C	-	-	T	C	C	C

A/G: Variation within reference genomes with X more predominant than Y (Same applies to others)

W,Y: Ambiguous nucleotides

-: Deletion

Table S6: Features of HPV 67 sequence alignment

Features / Sample ID	Position range	Length
Reference	6634-6718	85
GCNM 035	24-107	84

Table S7: Features of HPV 70 sequences alignment

Features / Sample ID	Position range	Length
Reference	6608-6703	96
GCP 083	26-120	95
GCNM 049	23-117	95
GCP 051	26-120	95
GCP 058	83-178	96

Table S8: Features of HPV 82 sequence alignment

Features / Sample ID	Position range	Length
Reference	6555-6704	150
GCP 095	2-142	141
